# 

**Published:** 1901-11-09

**Authors:** 


### NOTICE TO CORRESPONDENTS.

All MSS., Letters, Books for Review, and other matters intended for the Editor should be addressed The Editor, "The Hospital," The Hospital Building, 28 & 29 Southampton Street, Strand, W.C.

The Editor cannot undertake to return rejected MS., even when accompanied by stamped directed envelope.

**Notice to Advertisers and Subscribers.**

All Advertisements, Orders for Copies of The Hospital, and Business Communications should be addressed to **THE MANAGER (Not to the Editor),** The Hospital, 28 & 29 Southampton Street, Strand, London, W.C.

The Cost of Subscription, post free, is as follows:—Per week, 2½d.; per quarter, 2s. 8d.; per half-year, 5s. 3d.; per annum, 10s. 6d. Foreign:—Per annum, 15s. 2d., payable, in all cases, in advance.

**NOTICE.—Vol. XXX. of "The Hospital" (April 1901, to September 1901), in handsome cloth binding can now be obtained at the office of this Journal, price, 7s. 6d. Cases for binding the Numbers contained in this Volume 1s. 6d. Index to Vol. XXX., 2½d., post free.**

**The Monthly Part for November is now ready. Price 6d., by post 8d.**

### BOOKS RECEIVED.

Messrs. Longmans, Green and Co.

"A Practical Guide to the Administration of Anæsthetics." By R. J. Probyn-Williams, M.D.

"Elementary Hygiene" (Section I.) By William S. Furneaux.

Florence White.

"Small-pox, its Prevention and Treatment."

Simpkin and Co.

"Pocket Handbook for Monthly Nurses." By Margaret Chenevy.

Vail and Co.

"Annual Report on the Sanitary Condition of Islington." By Alfred Edwin Harris, Medical Officer of Health.

S. W. Partridge and Co.

"The Zenana, or Woman's Work in India." Vol. VIII.

"Surgeons and their Wonderful Discoveries." By F. M. Holmes.

The Department of Agriculture, N.W.T.

"Annual Report of the Department of Agriculture of the North-West Territories for 1900."

Argus Company, Capetown.

"Imperial Administration in South Africa during a Quarter of a Century." By the Viscount de Matalha.

Ville de Buénos-Ayres.

"Annuaire Statistique de la ville de Buénos-Ayres."

Hudson and Son, Birmingham.

"Annual Report on the Health of the City of Birmingham." By Alfred Hill, M.D., F.R.S.E., Medical Officer of Health.

Daniel and Co., St. Leonards.

"Annual Report on the Health of the Borough of Hastings." By A. Scarlyn Wilson, M.A., M.B., D.P.H., Medical Officer of Health.

Robertson and Gray, Coventry.

"Annual Report on the H-alth of the City of Coventry." By E. Hugh Snell, M.D., B.Sc.Lond., F.R.S.Ed., Medical Officer of Health.

John Wright and Co.

"First Aid Diagrams for the use of Lecturers."

### The Student of Medicine.

### APPOINTMENTS.

**D. V. M. Adams**, M.B., B.S.Edin., appointed Assistant Medical Officer to the Birmingham Parish Infirmary. **T. R. W. Armour**, M.B., appointed House Surgeon to the Hospital for Women, Liverpool. **J. B. C. Brockwell**, M.R.C.S.Eng., L.R.C.P.Lond., appointed Second Assistant Medical Officer to the Poplar and Stepney Sick Asylum District. **R. L. Brown**, L.R.C.P., L.R.C.S.Edin., L.F.P.S.Glasg., appointed Health Officer for the Fern Tree Gully Shire, Victoria. **Gordon Calthorp**, M.B., B.C.Cantab., appointed Certifying Factory Surgeon for the Wells District of Norfolk. **Charles Coles**, M.D., appointed Medical Officer of Health for the Bicester, Chipping Norton, Henley, Thame, Witney, and Woodstock Rural and Urban Districts, Hambledon, Headington, and Long Crendon Rural District, and Wheatley Urban District. **C. R. H. Crawford**, M.R.C.S., L.R.C.P.Lond., appointed Medical Officer of the Workhouse of the Tonbridge Union. **William Cumming**, M.D., appointed Health and Medical Officer at Cairns, Queensland. **A. Cutfield**, B.Sc.Lond., M.R.C.S.Eng., appointed District Medical Officer of the Ross Union. **Arthur E. B. Forster**, M.B. Melb., appointed Medical Superintendent at the Kyneton Hospital, Victoria. **C. G. Gibson**, M.B., C.M.Edin., reappointed Medical Officer of Health for the Broadwoodwiger and Launceston Rural Districts. **C. Hadley**, L.R.C.P.I., M.R.C.S.Eng., appointed District Medical Officer of the Nuneaton Union. **Francis W. E Hare**, M.D.Durh., appointed Medical Superintendent of the Diamantina Hospital for Chronic Diseases, Queensland. **C. O. Hawthorne**, M.D., M.R.C.P., appointed Assistant Physician to the North-West London Hospital. **A. G. Henry**, M.B., M.Ch.Syd., appointed Medical Superintendent at the Coast Hospital, Little Bay, New South Wales. **E. B. H. Hughes**, L.R.C.P., L.R.C.S.Edin., appointed Assistant Medical Officer to the Swansea Union Workhouse and Infirmary. **George H. Lancashire**, M.R.C.S.Eng., L.R.C.P.Lond., M.D.Brux., appointed Assistant Physician to the Manchester and Salford Hospital for Skin Diseases. **J. Marshall**, M.B. C.M Glasg., appointed District Medical Officer of the Halifax Union. **C. P. Mathew**, L.R.C.P.Lond., M.R.C.S.Eng., appointed District Medical Officer of the Beaminster Union. **E. J. F. Moore**, M.R.S.C.Eng., L.R.C.P.Lond., appointed Medical Officer to the West Street Workhouse, Hackney. **W. H. Savery**, L.S.A., appointed District Medical Officer of the Grimsby Union. **John W. L. Spence**, L.R.C.P., L.R.C.S.Edin.&Glasg., appointed Clinical Assistant to the Electrical Department of the Royal Infirmary, Edinburgh. **Hedley Terrey**, M.B., appointed Government Medical Officer and Vaccinator at Kiama, New South Wales. **A. H. Turner**, L.S.A., appointed Medical Officer of Health for the Beaconsfield Urban District.

ASSISTANT MEDICAL SUPERINTENDENT, &c.

